# Comparison of the hemodynamic response of dexmedetomidine versus additional intravenous lidocaine with propofol during tracheal intubation: a randomized controlled study

**DOI:** 10.1186/s12871-021-01484-6

**Published:** 2021-10-30

**Authors:** Rattaphol Seangrung, Koravee Pasutharnchat, Subundit Injampa, Sirima Kumdang, Rojnarin Komonhirun

**Affiliations:** Department of Anesthesiology, Faculty of Medicine, Ramathibodi Hospital, Mahidol University, 270 Rama VI Road, Ratchatewi, Bangkok, 10400 Thailand

**Keywords:** Dexmedetomidine, Lidocaine, Propofol, Hemodynamic response, Intubation

## Abstract

**Background:**

Laryngoscopy and tracheal intubation are strong stimuli that cause a reflex increase in blood pressure (BP), heart rate (HR), and serum catecholamine level. These can lead to myocardial infarction or cerebrovascular accidents. The purpose of this study is to compare the efficacy of dexmedetomidine and lidocaine combined with propofol in attenuating the hemodynamic response following laryngoscopy and endotracheal intubation.

**Methods:**

This study was a randomized controlled study and adhered to the CONSORT guidelines. One-hundred and six patients undergoing elective general anesthesia with endotracheal intubation were divided randomly into two groups. Group D received dexmedetomidine (1 μg kg^− 1^) before induction. Group LP received lidocaine (1.5 mg kg^− 1^) before induction with additional propofol (0.5 mg kg^− 1^) before laryngoscopy. The primary endpoint was hemodynamic including systolic (S) BP, diastolic (D) BP, mean arterial blood pressure (MAP) and HR measured before and after induction and ≤ 10 min after intubation. Secondary outcome was complications/adverse effects.

**Results:**

After induction, the mean SBP, DBP, MAP and HR decreased significantly from baseline in both groups except for mean HR in group LP at 1 min. Differences in mean values of SBP, DBP, and MAP were significantly lower in group D after intubation at 4–10 min (*P* <  0.05). Group LP had a non-inferior effect in blunting BP at all time points except 1 and 2 min after induction, and 2 min after intubation. The mean difference in HR in group D was significantly lower than that in group LP at all time points (*P* <  0.001). Group D had significantly more episodes of bradycardia (18.87% vs. 0%, *P* = 0.001) and hypotension (52.83% vs. 15.09%, *P* < 0.001) than did group LP.

**Conclusion:**

Lidocaine (1.5 mg kg^− 1^) with additional propofol (0.5 mg kg^− 1^) had a non-inferior effect compared with dexmedetomidine (1 μg kg^− 1^) in attenuating the hemodynamic response following laryngoscopy and endotracheal intubation, and had fewer adverse effects.

**Trial registration:**

Thai Clinical Trial Registry, (TRTC20190206002). Retrospectively registered 4 February 2019.

## Background

General anesthesia requires laryngoscopy and intubation, which can result in increased plasma concentrations of catecholamines [1–3]. An inadequate response to blunt stress can lead to hypertension and dysrhythmias, which can result in myocardial infarction or cerebrovascular accidents [4, 5].

Several agents have been employed to attenuate hemodynamic stress responses: dexmedetomidine, lidocaine, fentanyl, esmolol, propofol, or volatile anesthetic agents. Dexmedetomidine is a highly selective agonist of α-2 adrenergic receptors. It has a sympatholytic effect through a reduction in the norepinephrine concentration. Dexmedetomidine use can result in a decrease in blood pressure (BP) and heart rate (HR), so suppression of airway and circulatory reflexes during laryngoscopy and intubation is appropriate, [6, 7] but bradycardia and hypotension have been reported [8, 9].

Lidocaine is an aminoethylamide and prototype of amide-based local anesthetics. Usually, it is administered via the intravenous route at 1.5 mg kg^− 1^ bodyweight for 3 min before intubation to suppress the hemodynamic response. However, such suppression is not complete and a spike in systolic blood pressure (SBP) at 1-min and 3-min intervals post-intubation has been reported [10, 11].

Propofol is another anesthetic drug administered via the intravenous route. An additional dose of propofol (0.5 mg kg^− 1^) before intubation can significantly improve intubation conditions without increasing hypotension risk [12]. However, a study focusing on administration of lidocaine combined with a low dose of propofol to prevent the stress response, and whether it is non-inferior to dexmedetomidine use in facilitating adequate control of hemodynamics during laryngoscopy and intubation, has not been done.

In this study we compare the hemodynamic response and adverse effects/complications of dexmedetomidine versus lidocaine with additional propofol, before during and immediately after intubation.

## Methods

### Design

The protocol for this prospective, randomized control study was approved (05–60-30) on 17 September 2017 by the Ethics Committee (Chairman: Assistant Professor Dr. Chusak Okascharoen) of the Faculty of Medicine of Ramathibodi Hospital (Mahidol University, Bangkok, Thailand). This trial is registered at Thai Clinical Trial Registry on the 4 February 2019 (http://www.clinicaltrials.in.th/TRTC20190206002) and adhered to the CONSORT statement. Written informed consent was obtained from all patients according to the local regulations and to the principles of Helsinki Declaration.

### Exclusion criteria

Patients with hypertension, coronary artery disease, history of atrial fibrillation or other arrhythmias, implanted pacemakers, used of the antiarrhythmic drugs or beta blockers, cerebrovascular disease, a full stomach, or scheduled to have emergency surgery, or who were pregnant were excluded.

### Study cohort

The present study was undertaken in Ramathibodi Hospital from September 2017 to September 2019. One hundred and eleven patients (18–65 years; bodyweight, 40–80 kg; body mass index, 18–24.9 kg m^− 2^) with American Society of Anesthesiologists (ASA) physical status I–II and Mallampati classification grade I–II scheduled for elective surgery under general anesthesia with endotracheal intubation formed the study cohort.

### Anesthetic procedure

All patients who completely signed the inform consent would be accounted for in this study. Patient who was screen failures would be defined as who did not meet all the inclusion criteria and/or met at least on exclusion criteria and the patients who wished for withdraw from the study before randomization. Next,all patients were assessed preoperatively. Preoperative premedication with anxiolytics was not undertaken. In the preoperative suite, parameters at baseline were documented. Patients were hydrated with Ringer’s lactate solution approximately 8–10 ml kg^− 1^ within 20 min and followed by a maintenance infusion. Subsequently, patients were divided randomly by a computer program into two groups. Group D received 1 μg kg^− 1^ of dexmedetomidine (Precedex®; 100 μg ml^− 1^) in 20 ml of physiological (0.9%) saline over 10 min before the induction of anesthesia (hereafter termed “induction”). Group LP received 1.5 mg kg^− 1^ of lidocaine (2% lidocaine hydrochloride injection, preservative-free) in 20 ml of 0.9% saline over 3 min before induction by a nurse anesthetist not involved in the study.

**Fig. 1:**
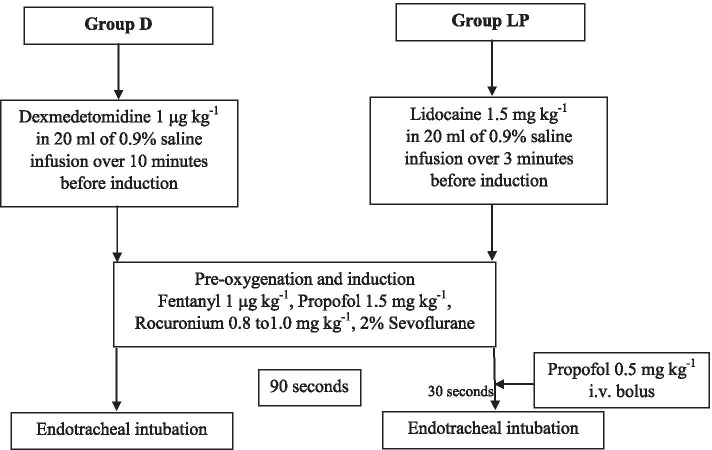
Study flow diagram

Upon arrival in the operating theatre, standard monitoring was recorded and patients underwent supplementation with 100% oxygen by a face mask at 6 l min^− 1^. In both groups, fentanyl (1.0 μg kg^− 1^, i.v.) was administered before induction. One minute after fentanyl administration, 1% propofol (Propofol-®Lipuro; 1.5 mg kg^− 1^) was administered to induce hypnosis until loss of consciousness was confirmed. Then, manual control of ventilation with 6 l min^− 1^ of 100% oxygen and 2% sevoflurane was carried out. Next, rocuronium (Esmeron®; 0.8–1.0 mg kg^− 1^) was injected. In group LP, an additional dose of propofol (0.5 mg kg^− 1^) was given 60 s after rocuronium administration. Ninety seconds after rocuronium administration, endotracheal intubation was undertaken by trainee anesthetists (Fig. 1). During the study, BP, HR, and oxygen saturation were monitored every minute. Another nurse not involved in the study recorded SBP, diastolic blood pressure (DBP), mean arterial pressure (MAP), and HR at preinduction, 1 min after induction, 2 min after induction, in the intubation phase, as well as 1, 2, 4, 6, 8, and 10 min after intubation. Hypotension was recorded if SBP < 90 mmHg or decreased > 20% compared with that at baseline. Bradycardia was noted if HR < 50 bpm. Rescue medication was given for hypotension (ephedrine, 6 mg, i.v.) and bradycardia (atropine, 0.6 mg, i.v.). However, to ensure the patient safety, patient allocation could be unmasked in occurrence of adverse events after study drugs administration. Anesthesiologists who involved in the patient care could stop the study drugs or adjust the infusion rate if the patients had the following conditions as severe hypotension, new onset of atrioventricular block, severe sinus bradycardia, or unexpected difficult airway management. These situations were documented in the record forms. For this the reasons led to protocol deviations. All of this patients were included in the intention to-treat analysis.

### Statistical analysis

Values for SBP, DBP, MAP, HR, and other continuous variables are given as the mean ± SD. Sex, ASA physical status, and other grouping variables are represented by numbers and percentages. A non-inferior margin of SBP ≥5 mmHg (determined from a study by Gulabani and colleagues [6]) in group LP (compared with group D) was used. Tests (Student’s *t*, Mann–Whitney, χ^2^) were used to compare characteristics between groups. Repeated-measures ANOVA was employed to assess changes over time within and between groups for SBP, MAP, DBP, and HR. SPSS 21.0 (IBM, Armonk, NY, USA) was used for data analyses. *P* < 0.05 was considered significant.

### Calculation of sample size

In this study, a non-inferior margin of SBP ≥5 mmHg at 3 min of post-intubation (determined from a study by Gulabani and colleagues [6]) was selected as important indicator for the calculation of the sample size. At the levels of α = 0.05 and β = 0.2, the sample size of each group was calculated to be at least 53 cases. Considering that approximately 5% of the patients would withdraw from the study.

## Results

**Fig. 2:**
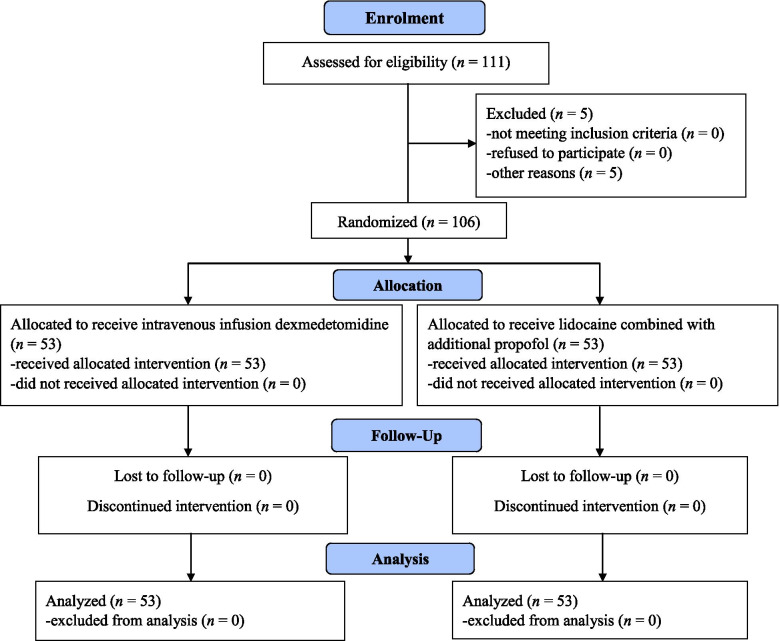
CONSORT flow chart

A CONSORT flowchart of our study is shown as Fig. 2. Between October 2017 and September 2019, 111 patients met the inclusion criteria and were recruited. Five patients dropped out owing to protocol violations and the anesthetic procedure was cancelled. Thus, 106 patients were evaluated. There were no intergroup differences in demographic characteristics (Table 1). The values of SBP, DBP, MAP, and HR at baseline were not significantly different (Table 2).

**Table 1:** Demographic data

	Group D, ***n*** = 53	Group LP, ***n*** = 53	***P*** value
Age (years)	43.49 ± 13.15	45.04 ± 11.58	0.522
Male/Female, *n*	11/42	13/40	0.643
BMI (kg m^− 2^)	22.49 ± 2.41	22.35 ± 2.63	0.775
ASA I/II, *n*	26/27	22/31	0.435
Mallampati score I/II/III, *n*	31/20/2	38/15/0	0.205
Time of intubation (seconds)	26.6 ± 9.03	30.19 ± 17.7	0.192
Attempt of intubation (times)	1 (constant)	1 (constant)	–
Laryngoscopic view 1/2, *n*	47/6	48/5	0.75

Data are presented as mean ± SD or number count

*ASA* American Society of Anesthesiologist physical status classification system, *BMI* Body mass index

**Table 2:** Blood pressure (mmHg) and heart rate (bpm) at different time points

	**DEX** (53)			**LP** (53)			Mean difference between groups (DEX-LP) (90%CI)	*P* value^‡^
	Mean (mmHg)	Mean difference within group		Mean (mmHg)	Mean difference within group			
**Systolic blood pressure**								
BL	128.25 ± 18.36			130.26 ± 15.01			−2.019 (−7.425–3.388)	0.537
		From BL	*P* value^†^		From BL	*P* value^†^		
BF-IND	124.83 ± 17.8	−3.415	0.060	132.28 ± 14.57	2.019	0.192	−7.453 [− 12.697 - (−2.208)]	0.020*
AFT-IND 1	115.09 ± 19.61	− 13.151	< 0.001*	115.53 ± 18.88	− 14.736	< 0.001*	− 0.434 (− 6.639–5.771)	0.908
AFT-IND 2	107.42 ± 19.05	− 20.830	< 0.001*	103.25 ± 17.85	− 27.019	< 0.001*	4.17 (− 1.781–10.121)	0.248
INT	121.28 ± 22.41	−6.962	0.015*	126.92 ± 26.33	− 3.340	0.389	−5.642 (− 13.523–2.239)	0.238
AFT-INT 1	117.66 ± 21.36	− 10.585	< 0.001*	121.4 ± 24.56	−8.868	0.014*	−3.736 (− 11.155–3.683)	0.405
AFT-INT 2	116.15 ± 44.22	−12.094	0.073	118.62 ± 20.64	− 11.642	< 0.001*	− 2.472 (− 13.597–8.654)	0.713
AFT-INT 4	102.47 ± 16.17	−25.774	< 0.001*	117.3 ± 21.2	− 12.962	0.001*	−14.83 [− 20.908 - (− 8.752)]	< 0.001*
AFT-INT 6	98.96 ± 13.97	− 29.283	< 0.001*	108.96 ± 14.47	−21.302	< 0.001*	− 10 [− 14.585 - (− 5.415)]	< 0.001*
AFT-INT 8	97.26 ± 16.56	− 30.981	< 0.001*	105.13 ± 13.45	−25.132	< 0.001*	− 7.868 [− 12.73 - (− 3.005)]	0.008*
AFT-INT 10	96.32 ± 13.74	− 31.925	< 0.001*	105.47 ± 14.01	− 24.792	< 0.001*	− 9.151 [− 13.625 - (− 4.677)]	0.001*
		Note: BL Mean 128.25, so 20%Mean≅25.65			Note: BL Mean 130.26, so 20%Mean≅26.05			
Result from repeated measures ANOVA (a within-subjects factor included 11 measurements) show no significant difference of the Between-groups effects (*P* = 0.036*)								
**Diastolic blood pressure**								
BL	77.7 ± 12.75			79.13 ± 9.92			−1.434 (− 5.117–2.249)	0.520
		From BL	*P* value^†^		From BL	*P* value^†^		
BF-IND	75.74 ± 11.92	−1.962	0.167	80.08 ± 9.74	0.943	0.447	− 4.34 [− 7.849 - (− 0.83)]	0.043*
AFT-IND 1	73.23 ± 12.6	− 4.472	0.025*	73.06 ± 14.51	−6.075	0.004*	0.17 (−4.21–4.55)	0.949
AFT-IND 2	68.25 ± 14.42	−9.453	< 0.001*	64.13 ± 14.58	− 15.000	< 0.001*	4.113 (− 0.562–8.788)	0.147
INT	78.53 ± 16.96	0.830	0.704	83.91 ± 19.77	4.774	0.105	−5.377 (− 11.316–0.561)	0.136
AFT-INT 1	77.43 ± 16.87	− 0.264	0.917	79.26 ± 17.06	0.132	0.959	− 1.83 (− 7.299–3.638)	0.580
AFT-INT 2	71.79 ± 14.25	− 5.906	0.011*	76.26 ± 14.72	− 2.868	0.162	− 4.472 (− 9.142–0.198)	0.115
AFT-INT 4	66.57 ± 14.1	− 11.132	< 0.001*	75.87 ± 18.44	− 3.264	0.192	−9.302 [− 14.594 - (− 4.009)]	0.004*
AFT-INT 6	62.45 ± 11.69	− 15.245	< 0.001*	68.66 ± 13.27	− 10.472	< 0.001*	− 6.208 [− 10.24 - (− 2.175)]	0.012*
AFT-INT 8	60.74 ± 13.61	− 16.962	< 0.001*	66.47 ± 13.09	− 12.660	< 0.001*	− 5.736 [− 10.039 - (− 1.432)]	0.029*
AFT-INT 10	61.42 ± 10.16	− 16.283	< 0.001*	67.11 ± 12.2	− 12.019	< 0.001*	−5.698 [− 9.318 - (− 2.079)]	0.010*
Result from repeated measures ANOVA (a within-subjects factor included 11 measurements) show no significant difference of the Between-groups effects (*P* = 0.045*)								
**Mean arterial pressure**								
BL	91.68 ± 13.93			92.85 ± 10.72			−1.17 (− 5.176–2.836)	0.629
		From BL	*P* value^†^		From BL	*P* value^†^		
BF-IND	89.6 ± 12.44	− 2.075	0.139	93.25 ± 10.89	0.396	0.745	−3.642 (− 7.41–0.127)	0.112
AFT-IND 1	83.96 ± 12.93	− 7.717	0.001*	83.21 ± 15.66	− 9.642	< 0.001*	0.755 (− 3.875–5.384)	0.787
AFT-IND 2	79.47 ± 14.26	− 12.208	< 0.001*	74.19 ± 15.5	− 18.660	< 0.001*	5.283 (0.482–10.084)	0.071
INT	90.74 ± 17.31	−0.943	0.689	96.64 ± 21.36	3.792	0.196	−5.906 (− 12.173–0.362)	0.121
AFT-INT 1	88.79 ± 17.27	− 2.887	0.265	91.26 ± 17.98	−1.585	0.558	−2.472 (− 8.154–3.211)	0.472
AFT-INT 2	81.77 ± 14.63	− 9.906	< 0.001*	88 ± 15.67	− 4.849	0.032*	− 6.226 [− 11.113 - (− 1.34)]	0.037*
AFT-INT 4	75.55 ± 13.95	− 16.132	< 0.001*	87.02 ± 18.49	− 5.830	0.031*	− 11.472 [− 16.753 - (− 6.191)]	< 0.001*
AFT-INT 6	72.21 ± 11.71	− 19.472	< 0.001*	79.45 ± 13.36	−13.396	< 0.001*	− 7.245 [− 11.296 - (− 3.195)]	0.004*
AFT-INT 8	71.87 ± 14.53	−19.811	< 0.001*	77.21 ± 13.48	− 15.642	< 0.001*	− 5.34 [− 9.858 - (− 0.821)]	0.053
AFT-INT 10	71.45 ± 10.46	− 20.226	< 0.001*	77.79 ± 12.27	− 15.057	< 0.001*	− 6.34 [− 10.015 - (− 2.664)]	0.005*
Result from repeated measures ANOVA (a within-subjects factor included 11 measurements) show no significant difference of the Between-groups effects (*P* = 0.035*)								
	**DEX** (53)			**LP** (53)			Mean difference between groups (DEX-LP) (90%CI)	*P* value^§^
	Mean (bpm)	Mean difference within group		Mean (bpm)	Mean difference within group			
**Heart rate**								
BL	74.42 ± 14.03			78.7 ± 13.55			− 4.283 (− 8.729–0.163)	0.113
		From BL	*P* value^†^		From BL	*P* value^†^		
BF-IND	66.43 ± 15.22	− 7.981	< 0.001*	79.47 ± 13.45	0.774	0.589	−13.038 [− 17.668 - (− 8.407)]	< 0.001*
AFT-IND 1	61.51 ± 13.69	− 12.906	< 0.001*	76.02 ± 13.36	− 2.679	0.137	− 14.509 [− 18.87 - (− 10.149)]	< 0.001*
AFT-IND 2	60.81 ± 13.74	− 13.604	< 0.001*	73.28 ± 11.73	− 5.415	0.002*	− 12.472 [− 16.59 - (− 8.353)]	< 0.001*
INT	70.53 ± 13.51	− 3.887	0.057	84.64 ± 16.12	5.943	0.009*	− 14.113 [− 18.909 - (− 9.318)]	< 0.001*
AFT-INT 1	72.92 ± 12.88	−1.491	0.448	82.62 ± 14.34	3.925	0.046*	−9.698 [− 14.093 - (− 5.303)]	< 0.001*
AFT-INT 2	71.11 ± 11.07	− 3.302	0.065	82.4 ± 13.83	3.698	0.065	−11.283 [− 15.322 - (− 7.244)]	< 0.001*
AFT-INT 4	69.58 ± 10.82	− 4.830	0.005*	80.43 ± 13.03	1.736	0.373	− 10.849 [− 14.711 - (− 6.987)]	< 0.001*
AFT-INT 6	67.11 ± 10.54	− 7.302	< 0.001*	78.58 ± 13.03	− 0.113	0.956	−11.472 [−15.293 - (− 7.651)]	< 0.001*
AFT-INT 8	66.49 ± 9.27	− 7.925	< 0.001*	76.57 ± 12.64	− 2.132	0.304	−10.075 [− 13.653 - (− 6.498)]	< 0.001*
AFT-INT 10	65.13 ± 9.86	− 9.283	< 0.001*	74.11 ± 12.37	− 4.585	0.022*	−8.981 [− 12.587 - (− 5.376)]	< 0.001*
Result from repeated measures ANOVA (a within-subjects factor included 11 measurements) show no significant difference of the Between-groups effects (*P* < 0.001*)								

Data are mean ± SD

*BL* Baseline, *BF-IND* Before induction, *AFT-IND n* After induction at *n* min, *INT* Intubation, *AFT-INT n* After intubation at *n* min

^*^ *P* – value < 0.05 (two-sided tests)

^†^ compared with “Baseline” value within individual groups using paired t-tests

^‡^ compared across groups at each time point using independent t-tests

**Fig. 3:**
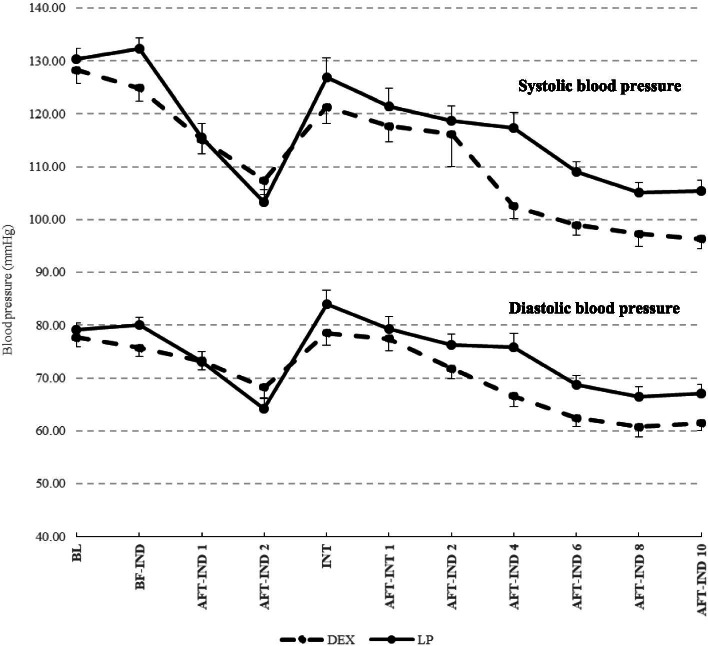
Mean of Systolic and Diastolic Arterial Blood Pressure for each time point (Dash line, DEX; Solid line, LP)

**Fig. 4:**
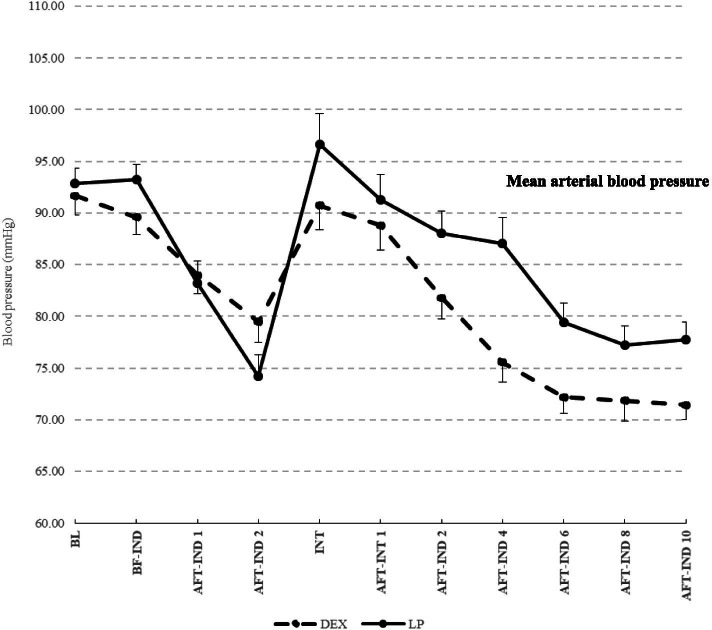
Mean of Mean arterial Blood Pressure for each time point (Dash line, DEX; Solid line, LP)

SBP, DBP, and MAP in group LP were decreased from baseline values more than those in group D at 1 min and 2 min after induction (SBP: − 14.73 and − 27.01 vs. − 13.15 and − 20.83 mmHg; DBP: − 6.07 and − 15.00 vs. − 4.47 and − 9.45 mmHg: MAP: − 9.64 and − 18.66 vs. − 7.71 and − 12.20 mmHg, respectively) but were not significantly different between groups (Figs. 3 and 4, Table 2). Simultaneously, in group LP, SBP at 2 min after induction decreased > 20% from that at baseline (− 27.01 mmHg; 20% of the mean SBP at baseline [130.26 mmHg] was 26.05 mmHg) but this value was not significantly different when compared with the SBP of group D (Table 2).

In the intubation period, only the SBP in group D was significantly lower than that at baseline (*P* < 0.05). The SBP, DBP, and MAP were not significantly different when compared between groups (Table 2).

For group D, the mean SBP, DBP, and MAP decreased significantly from baseline at 4–10 min after intubation (*P* < 0.001), and the mean differences were significantly lower than those in group LP. Meanwhile, the SBP decreased > 20% from baseline in group D at 4–10 min after intubation (20% of the mean SBP at baseline [128.25 mmHg] was 25.65 mmHg) (Table 2).

**Fig. 5:**
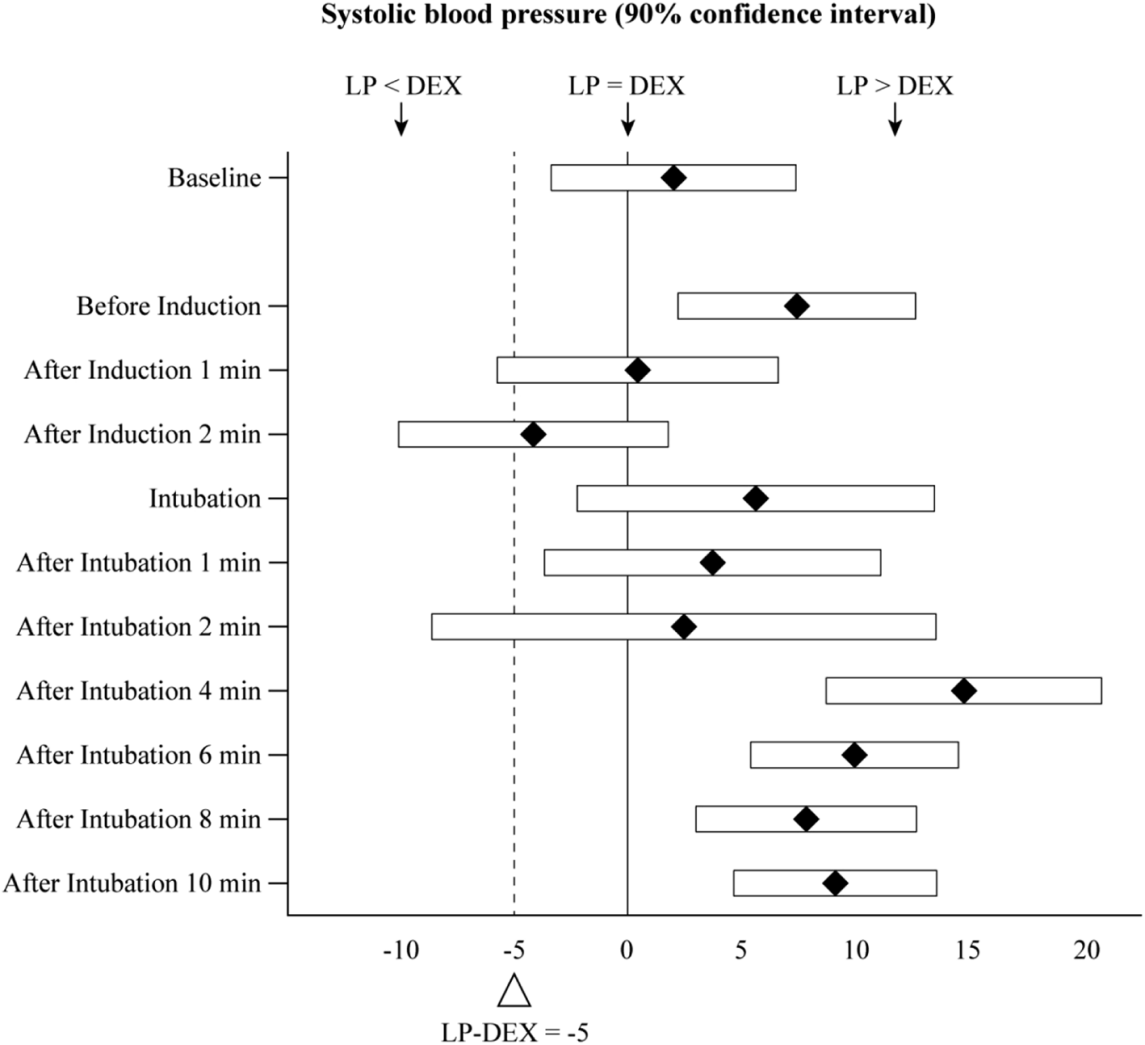
Forest plot showing the superiority and non-inferiority hypothesis testing for all-time systolic blood pressure differences between groups

A forest plot showing the difference in SBP between groups is shown as Fig. 5. A non-inferior margin of SBP ≥5 mmHg (determined from a study by Gulabani and colleagues^6^) in group LP (compared with group D) was used in our study. Group LP had a non-inferior effect in blunting BP at all time points except 1 min and 2 min after induction, and 2 min after intubation.

**Fig. 6:**
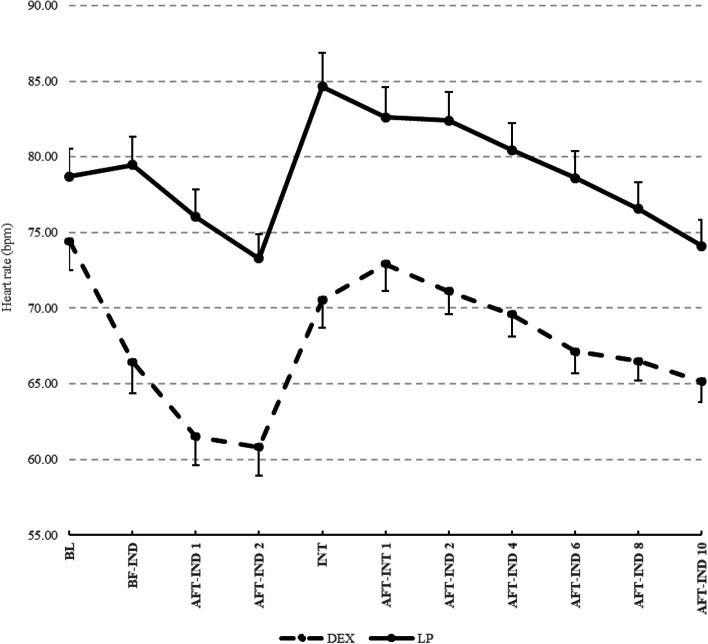
Mean of Heart rate for each time point (Dash line, DEX; Solid line, LP)

The mean difference in HR in group D was significantly lower than that in group LP in each period from preinduction until 10 min after intubation (*P* < 0.001) (Fig. 6, Table 2). Nevertheless, in group LP, the HR decreased significantly from baseline to 2 min after induction (73.28 ± 11.73 bpm; mean difference, − 5.41; *P* < 0.05) and increased significantly from baseline to the intubation phase (84.64 ± 16.12 bpm; mean difference, 5.94; *P* < 0.05) and 1 min after intubation (82.62 ± 14.34 bpm; mean difference, 3.92, *P* < 0.05) (Table 2).

Bradycardia and hypotension occurred in group D significantly more often than in group LP. The prevalence of bradycardia in group D was 18.87%, whereas bradycardia did not occur in group LP. The prevalence of hypotension in group D was 52.83%, whereas it was 15.09% in group LP (Table 3).

**Table 3:** Adverse effects or complications

Adverse effects or complications	Group		***P*** value
	D (***n*** = 53)	LP (***n*** = 53)	
**Bradycardia (**Number [%]**)**	10 [18.87]	0 [0.00]	0.001*
**Hypotension (**Number [%]**)**	28 [52.83]	8 [15.09]	< 0.001*

*P < 0.05

## Discussion

Laryngoscopy and endotracheal intubation are strong stimuli. They can cause a sympathomimetic response that manifests as hypertension and dysrhythmias that can lead to myocardial infarction and cerebrovascular accidents [4]. Reddy and colleagues [8] and Yildiz and coworkers [9] found that dexmedetomidine could blunt the sympathomimetic response to laryngoscopy and endotracheal intubation, but also resulted in hypotension and bradycardia. In the present study, dexmedetomidine had similar effects to those recorded in the studies by Reddy and colleagues [8] and Yildiz and coworkers [9]. That is, SBP and MAP decreased after induction, and decreased significantly 4–10 min after intubation. SBP decreased > 20% from that recorded at baseline after intubation, which denoted hypotension. The HR decreased significantly during induction as well as 4–10 min after intubation when compared with the HR at baseline. In the intubation period, only SBP changed significantly from baseline: it decreased ≤20% from that at baseline.

From the previous study, Dashti and colleagues [13] compared hemodynamic alterations following tracheal intubation with the GlideScope® video-laryngoscope (GVL) and the Macintosh direct laryngoscope (MDL). It has been shown to increase of blood pressure and heart rate during oropharyngeal, laryngeal and tracheal stimulation. Then, both blood pressure and heart rate would return to pre-intubation values at 3 and 4 min after intubation, respectively. Therefore, these changes were transient and returned to the baseline levels within 5 min after intubation because of discontinuing airway stimulation. Our study demonstrated that dexmedetomidine elicited extreme alterations in hemodynamics 4–10 min after intubation. It was probably due to a result of pharmacodynamic interactions of dexmedetomidine with anesthetics, sedatives, and opioids might lead to the decline of blood pressure and heart rate after 4 min. Thus, co-administration of dexmedetomidine with propofol or opioids can lead to hemodynamic instability. However, Cabrini and colleagues [14] published the recent systematic review of randomized controlled trials that intravenous sedation with dexmedetomidine alone resulted in safety and a few adverse events in awake fiberoptic intubation. Each of the techniques for intubation would stimulate the different hemodynamic responses. It can be seen that the administration of anesthesia should be adjusted according to the intensity of the stimulus so it can lead to a reduction in complications. Wilson and colleagues demonstrated that lidocaine alone can reduce BP but not the chronotropic response to laryngoscopy and tracheal intubation [11]. Propofol has myocardial-depressant effects and decreases systemic vascular resistance in a dose-dependent manner. Kwon and colleagues [12] found that an additional dose of propofol (0.5 mg kg^− 1^) before intubation could improve intubation conditions significantly. In our study, lidocaine (1.5 mg kg^− 1^, i.v.) given before induction combined with an additional dose of propofol (0.5 mg kg^− 1^) given 30 s before intubation led to all parameters having similar trends to those elicited using dexmedetomidine. SBP, DBP, and MAP in group LP decreased markedly after induction, and decreased after intubation. Furthermore, we showed that SBP at 2 min after induction decreased > 20% from that recorded at baseline. Hendrickx and colleagues [15] and Woods and collaborators [16] reported that propofol and lidocaine (i.v.) had a synergistic effect that led to a significant drop in BP after induction: we postulate that hypotension was due to this synergistic effect. However, there was no significant change in SBP, DBP, or MAP in the intubation phase when compared with that at baseline. Furthermore, the HR in group LP decreased slightly after induction but increased in an acceptable range upon and after intubation.

Dexmedetomidine use has been reported to increase the risk of bradycardia and hypotension [8, 9]. The prevalence of hypotension and bradycardia in group D was 52.83 and 18.87%, respectively. Yildiz and coworkers [9] used atropine (0.5 mg, i.m.) as premedication 30 min before infusion of dexmedetomidine (1.0 μg kg^− 1^) for tracheal intubation, and the prevalence of hypotension (16%) and bradycardia (4%) was lower than that in our study. However, a study using lidocaine with an additional low dose of propofol before induction to blunt the cardiovascular reflex has not been caried out. The prevalence of hypotension and bradycardia in group LP was 15.09 and 0%, respectively, which were lower than those in group D and statistically significant. Our study had some limitations. First, our study included only healthy, non-obese, and young patients of ASA physical status I–II. Second, we did not measure the depth of anesthesia during the procedure in either group owing to a lack of facilities. An inadequate depth of anesthesia can lead to an increase in the hemodynamic response to intubation. Third, our study cohort was healthy, so we did not use invasive monitoring. The latter might be more sensitive and provide a more accurate measurement of hemodynamic change, including intravascular postoperative blood glucose was not analyzed Finally, anesthesiologists were not blinded to the group of study subjects. However, they would follow to the study protocol.

Further study should include obese cases patients with ASA physical status >II or history of atrial fibrillation or other arrhythmias, implanted pacemakers, used of the antiarrhythmic drugs or beta blockers. In addition, a study in cost-effectiveness should be conducted.

Our data suggest that lidocaine with propofol could be used as an alternative to a direct sympathomimetic response from laryngoscopy and laryngeal intubation. Moreover, administration of lidocaine and propofol is less expensive than dexmedetomidine administration and associated with fewer complications.

## Conclusions

Lidocaine (1.5 mg kg^− 1^) with additional propofol (0.5 mg kg^− 1^) had a non-inferior effect compared with that of dexmedetomidine (1 μg kg^− 1^) for attenuating the hemodynamic response following laryngoscopy and endotracheal intubation only in healthy patients, and was also associated with fewer complications.

## Data Availability

The trial protocol, datasets used and/or analysed of this study are available from the corresponding author on reasonable request.

## Abbreviations

BP: Blood pressure
HR: Heart rate
SBP: Systolic blood pressure
DBP: Diastolic blood pressure
MAP: Mean arterial pressure
ASA: American Society of Anesthesiologists
